# Indian Ethnomedicinal Phytochemicals as Promising Inhibitors of RNA-Binding Domain of SARS-CoV-2 Nucleocapsid Phosphoprotein: An *In Silico* Study

**DOI:** 10.3389/fmolb.2021.637329

**Published:** 2021-07-02

**Authors:** Sankar Muthumanickam, Arumugam Kamaladevi, Pandi Boomi, Shanmugaraj Gowrishankar, Shunmugiah Karutha Pandian

**Affiliations:** ^1^ Department of Bioinformatics, Alagappa University, Karaikudi, India; ^2^ Department of Animal Science, School of Life Sciences, Bharathidasan University, Tiruchirappalli, India; ^3^ Department of Biotechnology, Alagappa University, Karaikudi, India

**Keywords:** ADMET profiles, medicinal plants, molecular docking, molecular dynamic simulation, nucleocapsid phosphoprotein, SARS-CoV-2

## Abstract

SARS-CoV-2, an etiological agent of COVID-19, has been the reason for the unexpected global pandemic, causing severe mortality and imposing devastative effects on public health. Despite extensive research work put forward by scientist around globe, so far, no suitable drug or vaccine (safe, affordable, and efficacious) has been identified to treat SARS-CoV-2. As an alternative way of improvising the COVID-19 treatment strategy, that is, strengthening of host immune system, a great deal of attention has been given to phytocompounds from medicinal herbs worldwide. In a similar fashion, the present study deliberately focuses on the phytochemicals of three Indian herbal medicinal plants *viz*., *Mentha arvensis*, *Coriandrum sativum*, and *Ocimum sanctum* for their efficacy to target well-recognized viral receptor protein through molecular docking and dynamic analyses. Nucleocapsid phosphoprotein (N) of SARS-CoV-2, being a pivotal player in replication, transcription, and viral genome assembly, has been recognized as one of the most attractive viral receptor protein targets for controlling the viral multiplication in the host. Out of 127 phytochemicals screened, nine (linarin, eudesmol, cadinene, geranyl acetate, alpha-thujene, germacrene A, kaempferol-3-O-glucuronide, kaempferide, and baicalin) were found to be phenomenal in terms of exhibiting high binding affinity toward the catalytic pocket of target N-protein. Further, the ADMET prediction analysis unveiled the non-tumorigenic, noncarcinogenic, nontoxic, non-mutagenic, and nonreproductive nature of the identified bioactive molecules. Furthermore, the data of molecular dynamic simulation validated the conformational and dynamic stability of the docked complexes. Concomitantly, the data of the present study validated the anti-COVID efficacy of the bioactives from selected medicinal plants of Indian origin.

## Introduction

Although a year has been completed since the unprecedented emergence of severe acute respiratory syndrome coronavirus (SARS-CoV-2), the pandemic menace prevails till date. The World Health Organization’s (WHO) Day-to-Day data till 4th February 2021 state that the morbidity rate of “coronavirus disease 2019” (COVID-19) across 220 countries is crossing 102.9 (102, 942, 987) million, and the rate of mortality is 2.29 (2,232,233 deaths). The ever-increasing infected victims as well as mortality rate alarms the dire need for early diagnosis and identification of a drug or vaccine to treat COVID patients. Owing to the lack of therapeutic choices, the WHO announces COVID-19 as a “public health emergency of international concern (PHEIC),” implying that this pandemic seeks orchestrated global action in all clinical aspects (de Wit et al., 2016; Wu C. et al., 2020a).

SARS-CoV-2 is a positive-sense single-stranded (+ss) RNA virus that belongs to the family Coronaviridae and genera *Betacoronavirus*. It infects a wide range of host, including human, cattle, pigs, cats, and birds. Particularly, in human, it causes various symptoms, from mild respiratory infections, fever, dyspnea, lung lesions (Li et al., 2005; Killerby et al., 2018), and enteric disease to severe life-threatening pneumonia (de Wit et al., 2016; Killerby et al., 2018; Phan et al., 2020a; Huang et al., 2020; Li et al., 2020; Parry, 2020; Riou and Althaus, 2020). As far as strategies of SARS-CoV-2 spread are concerned, the significant virulence traits including immune evasion, replication inside host, and transmission from human-to-human are the major barriers for clinicians and other healthcare workers to treat and prevent COVID-19 (Chan et al., 2020; Li et al., 2020; Prompetchara, et al., 2020).

In general, the viral replication inside the host cell involves the synthesis of proteins, namely, envelope (E), membrane/matrix (M), spike (S), and nucelocapsid phosphoprotein (N) (Brian and Baric 2005). Accordingly, in the recent days, the E, M, S, and N proteins have been targeted for antiviral drug and vaccine designing investigations. However, any mutation in the outer membrane proteins *viz.*, S, E, and M proteins aid SARS-CoV-2 to gain drug resistance (Benvenuoto et al., 2020; Phan 2020b; Dawood, 2020; Ou et al., 2020; Pachetti et al., 2020; Yin, 2020). Therefore, the N protein has particularly been considered as an attractive drug target (Wu F. et al., 2020b; Zhou et al., 2020).

The N protein of SARS-CoV-2 is a multifunctional protein chiefly involved in viral replication (Chang et al., 2014), virulence, immunogenicity (Burbelo et al., 2020; Randad et al., 2020), and pathogenesis (Yasui et al., 2008; Gao et al., 2020). The C-terminal domain (CTD) of the N protein binds with the M protein through dimerization and creates a physical link between the viral genome and its envelope, which thereby forms the helical ribonucleoprotein (RNP) complex. This complex not only renders protection to viral genome but also modulates the host intracellular machinery, and consistently plays a regulatory role throughout the viral life cycle (Masters et al., 1990; Narayanan et al., 2003; Chang et al., 2009). Earlier studies have robustly demonstrated the contribution of the N protein in host–pathogen interactions by regulating host cell cycle, apoptosis, and actin reorganization (Hsieh et al., 2005; Surjit et al., 2006). In addition, the viral N protein inhibits interferon-β and thus facilitates SARS-CoV-2 to evade the host immune response (Kopecky-Bromberg et al., 2000; Lu et al., 2011). Therefore, such a protein that majorly contributes to the viral replication and immune evasion could be a promising target to develop therapeutic countermeasures in controlling SARS-CoV-2 and infection-mediated further havoc.

As traced to antique Indian traditional medicinal system, the consumption of plant and plant-derived natural products has shown efficient therapeutic effects against various health ailments (Alagu Lakshmi et al., 2020; Muthuramalingam et al., 2020; Vellingiri et al., 2020; Gowrishankar et al., 2021). Notably, consumption of herbal plants has been a well-recognized home remedy for common cold (Alagu Lakshmi et al., 2020; Muthuramalingam et al., 2020; Vellingiri et al., 2020; Gowrishankar et al., 2021). Against common cold, a wide spectrum of herbs with proven medicinal benefits have been used in the traditional home remedy that reinforce the immune system (Lin et al., 2014; Wang and Liu 2014; Ganjhu et al., 2015). Based on this, three herbal plants *viz*., *Mentha arvensis*, *Coriandrum sativum*, and *Ocimum sanctum* were considered in the present study.

*Mentha arvensis* L., an aromatic plant popularly known as menthol mint and kitchen herb (in India; The wealth of India, 2003), holds not only medicinal values but also clutches varied industrial applications *viz*., flavorings, food, confectionary, cosmetic, perfumery, and pharmaceutics (Kumar et al., 2012; Lal et al., 2020). *M. arvensis* is a natural antioxidant (Kumar and Chattopadhyay, 2007), and it has been reported to exhibit antimycotic efficacy (Yadav et al., 2006). The mint leave juice also displayed diversified health benefits as it has been administered against liver and spleen disease, diarrhea, dysentery, indigestion, asthma, and jaundice. It has been a traditional remedy for rheumatic pains, arthritis, and inflamed joints (Salin et al., 2011; de Sousa Guedes et al., 2016; de Sousa Guedes and Souza, 2017). *C. sativum*, exhibits myriad pharmaceutical potentials (*viz*., antidiabetic, antiseptic, anti-inflammatory, antihypertensive, anxiolytic, antimicrobial, anti-cancerous, antimutagenic, diuretic, cognition improvement, and antioxidant) (Hussain et al., 2018; Kačániová et al., 2020), and its inhibitory efficacy against angiotensin-converting enzyme has been envisaged as the most significant action mechanism against COVID-19 (Khan and Kumar, 2019; Wei et al., 2019; Vellingiri et al., 2020). *O. sanctum* (tulsi) has been the holy herb with innumerable medicinal/health benefits, deployed since ancient period. It has been well demonstrated for multifaceted therapeutic propensity *viz*., anti-inflammatory, antidiabetic, immunomodulatory, antifertility, anticancer, cardio and hepatoprotective, antiviral, antifungal, and antibacterial efficacies (Seth and Sharma, 2004; Prakash and Gupta, 2005; Mallikarjun et al., 2016; Yamani et al., 2016; Jamshidi and Cohen, 2017; Mousavi et al., 2018). Most importantly, tulsi leaves have been proven to show beneficial effects against bronchitis and pyrexia through boosting/strengthening cellular as well as humoral immune responses (Mukherjee et al., 2005). Although a few reports have documented the plausible anti-COVID efficacy of *O. sanctum* as it targets the main protease of SARS-CoV-2, the efficacy of three selected plants against SARS-CoV-2 N protein has not been explored so far. Therefore, in the current investigation, the phytochemicals of these herbal plants (*M. arvensis*, *C. sativum*, and *O. sanctum*) were analyzed for their potential to inhibit the N protein through an *in silico* approach.

## Materials and Methods

### Protein Selection and Active Site Prediction

The crystal structure of the SARS-CoV*-*2 nucleocapsid phosphoprotein essential for virion formation and replication (PDB ID: 6ZCO; 1.36 Å) was retrieved from the RCSB Protein Databank (https://www.rcsb.org/). Based on the resolution (1.36 Å), stable atomic orientation, and CTD of the N protein crystal structure, 6ZCO was selected in the present study. All crystalline water molecules and bound ligand molecules were removed, and the polar hydrogen and Gasteiger charges to protein structures for the docking simulation were also assigned, as described earlier by Afriza et al. (2018).

### Ligand Selection and Preparation

The chemical structure of phytocompounds was obtained from the Pubchem database (https://pubchem.ncbi.nlm.nih.gov/) in .sdf (structure date file) format. Then the file format was converted to PDB (Protein Data Bank) coordinate file format using the Open Babel (http://openbabel.org) (Rolta et al., 2020).

### Molecular Docking

Molecular docking studies were conducted using AutoDock Vina in order to predict the accuracy of binding affinity as well as ligand-binding poses into protein active sites. Initially, both the ligand and receptor were preprocessed by adding the hydrogen, to assign the charge particle, and to remove the unwanted water molecules and heteroatom, and file format conversion was done by AutoDockTools. Then the grid map was defined to the active site (Ala264, Val270, Phe274, Arg277, Gla281, Phe286, and Gly284) of the receptor, and the grid box dimension was set as 20 × 20 × 20. The default scoring function of AutoDock Vina was used to calculate the docking score, and the lowest binding energy docking poses were selected for further interaction analysis as described in an earlier study by Chen et al. (2018). Discover Studio 3.5 is used to analyze the binding pose 2D and 3D interaction analysis of the protein–ligand complexes (Shivanika et al., 2020).

### ADME Prediction

The top hit compounds obtained through molecular docking studies were further screened based on their ADME (absorption, distribution, metabolism, and excretion) properties, physicochemical properties (Lipinski’s rule of five principles), pharmacokinetics (Pks), and drug-likeness properties using the Molinspiration and AdmetSAR servers (Isa et al., 2018).

### Molecular Dynamic Simulation

The obtained docking results of the best docked complexes were further subjected to molecular dynamic (MD) simulation using the GROMACS 4.5.5 package with the GROMOS53a6 force field for all atoms to get a protein topology parameter. The PRODRG web server was used to analyze the topology and force filed parameter of the ligand (Zheng et al., 2014). The protein–ligand complexes were solvated in a cubic box with the water model of SPC216 and neutralized by adding -Cl counter ions. Then the energy minimization of the system was performed by using the steepest descent algorithm. To equilibrate the system with constant volume and temperature from 300 K for 100 ps, NVT ensembles followed by the NPT ensembling at a constant temperature and constant pressure for 300 K for 1 bar. Finally, MD simulations were conducted for 50 ns (Ul Qamar et al., 2019). Root mean square deviation (RMSD), hydrogen bond analysis, radius of gyration (Rg), potential energy, root mean square fluctuations (RMSFs), secondary structure analyses, and SASA were done using GROMACS. XM Grace software was used to analyze the plot of RMSD, RMSFs, hydrogen bonds, *etc*. (Muthumanickam et al., 2020).

## Results and Discussion

Given the prominence that boosting/strengthening the immune status of an individual would be a convincing alternative to prevent COVID infectivity, we deliberately investigated three selective medicinal herbs (*viz*., *Mentha arvensis*, *Coriandrum sativum*, and *Ocimum sanctum*) against one of the most important structural proteins named nucleocapsid phosphoprotein, which is a least variable and highly conserved structure of CoV (Lin et al., 2014). Indian traditional knowledge system has a historical background with proof of concept toward curing effects against common cold. In view of that, during this COVID-19 pandemic, Ministry of AYUSH, Government of India has identified and listed diverse medicinal shrubs and herbs employed in-house as home remedies with proven efficacy to strengthen the respiratory tracks and immune system.^6^ Most evidently, the WHO has estimated that nearly 80% of the population in underdeveloped countries depend chiefly on traditional medicines against COVID-19. On the view of tradition-based phyto-immune boosters, the WHO has enlisted nearly 21,000 global plants of therapeutic potential; among which, around 2,500 varieties were of Indian origin.^33^

Indian traditional system strongly relies on the quote “Food as Medicine,” and the other armors such as balanced diet and proper physical exercise further immunize the system. Based on these Indian naturopathic values, the patients affected with respiration illness were recommended for herbal steam inhalation therapy to subset the symptoms (Amini et al., 2017; Singh et al., 2017). In substantiation with the current study, earlier reports by Alagu Lakshmi et al. (2020) had signified the scientific merit toward deploying complementary herbal medicines against COVID-19. Similarly, in an earlier report by Singh et al. (2017), they denoted the speedy improvement of patients infected with common cold viral infection upon neti treatment along with vitamins as well as minerals (Amini et al., 2017; Singh et al., 2017). In an earlier study by our group, we have demonstrated the promising effects of phytochemicals from traditional Indian herbal steam inhalation therapy against COVID-19 through an *in silico* approach (Gowrishankar et al., 2021). Therefore, it is anticipated that phytochemicals might possibly set forth an initial developmental step for combinatorial naturopathic therapy either as an antiviral agent or as an immune booster in order to effectively manage COVID-19.

Unlike the other studies that target different structural proteins of SARS-CoV-2, in the present study, we chose a most important viral structural protein, nucleocapsid phosphoprotein, as it is highly abundant and least variable as well as highly conserved in CoV (Lin et al., 2014). The three domains of the N protein holds three different roles *viz*., N-terminal binds RNA, C-terminal aids in oligomerization, and central Ser/Arg rich linker helps in phosphorylation reactions (Chang et al., 2013; Yadav et al., 2020). This strong binding of the N protein with the RNA genome creates ribonucleoprotein complex, which exclusively triggers the production of virion core and RNA-dependent RNA synthesis for replication of virus (McBride et al., 2014; Cong et al., 2020). In addition to it, N-proteins have been investigated to uphold regulatory role during infection with host, starting from actin filament reorganization to apoptosis (Surjit et al., 2006; Du et al., 2008). As the N protein involves in the replication, transcription, and viral genome assembly, it could be an attractive drug target of SARS-CoV-2 in controlling the viral multiplication in host (Yadav et al., 2020). In par with the current study, a very recent study by Yadav et al. (2020) has emphasized that the inhibition of the N protein would be a convincing approach in treating the viral disease progression. Therefore, in the present study, we intentionally made an effort to virtually substantiate the antiviral efficacy of three AYUSH, GoI enlisted immune booster Indian herbs’ (*viz*., *Mentha arvensis*, *Coriandrum sativum*, and *Ocimum sanctum*) associated compounds against the N protein of SARS-CoV-2 through an *in silico* approach.

### Molecular Docking Studies

In order to identify the potential drug candidates for managing COVID-19, molecular docking analysis was performed for 127 phytoconstituents from three selected medicinal plants against the active site of SARS-CoV-2 RNA binding domain of nucleocapsid phosphoprotein. The results revealed that most of the phytoconstituents interacted with target protein efficiently. Further, the phytoconstituents with the highest docking affinity were assigned as potential small molecules, and their interaction analysis was studied in detail. The overall binding affinity of 127 phytoconstituents toward the target protein is tabulated in Supplementary Table S1.

### Binding Mode of Phytoconstituents From *Mentha arvensis* Against the N Protein

**FIGURE 1:**
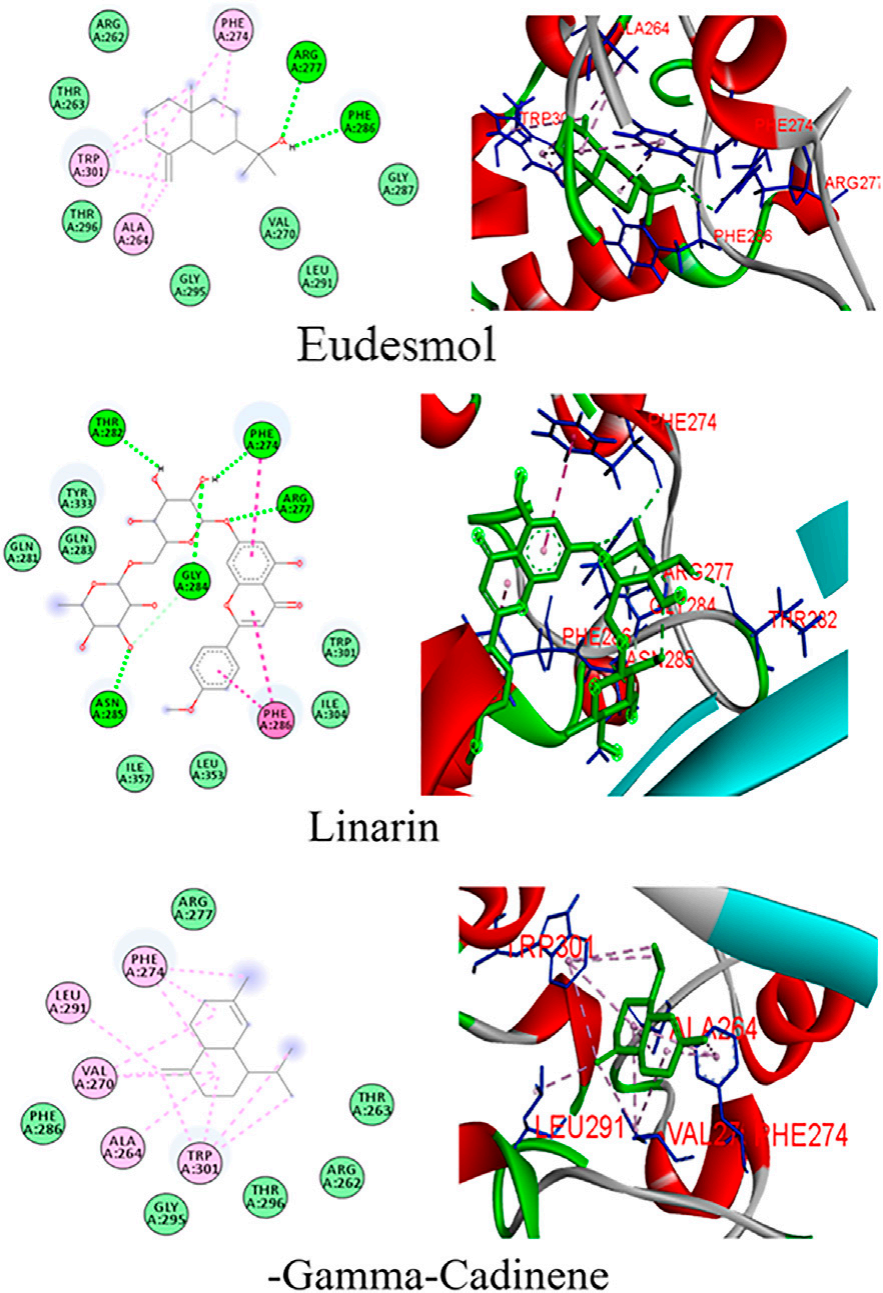
Binding patterns of the top phytoligands from *Mentha arvensis* against the N protein.

Docking simulations of major phytocompounds of *Mentha arvensis* against the N protein using ADT showed eudesmol as a top hit, exhibiting the highest docking score of −10.1 kcal/mol, followed by the phytochemicals linarin (−8.4 kcal/mol) and (−)−gamma−cadinene (−7.5 kcal/mol) (Table 1). The results indicated that out of sixty-six small molecules screened virtually, eudesmol, linarin, and (−)−gamma−cadinene possess the strong interactions with the N protein by executing greatest binding affinity. The amino acid residues of the N protein involved in hydrogen bond, hydrophobic, and electrostatic interactions with these ligands were also observed through docking analysis using AutoDock Vina (Figure 1).

**TABLE 1:** List of top three hit phytochemicals from each of the three selected plants (*Mentha arvensis*, *Coriandrum sativum*, and *Ocimum tenuiflorum*) along with their binding energy and interaction residues against the N protein as predicted through molecular docking.

Compound name	Binding affinity	Interaction residues
Interaction diagram of *Mentha arvensis* [mint]		
Eudesmol	−10.1	ALA264, PHE274, ARG277, PHE286, TRP301
Linarin	−8.4	PHE274, ARG277, THR282, GLY284, PHE286
(−)−Gamma-cadinene	-7.5	ALA264, VAL270, PHE271, LEU291, TRP301
*Coriandrum sativum* [coriander]		
(+)−Germacrene	−7.1	PHE314, TYR333
Alpha-thujene	−6.5	ALA264, VAL270, PHE286, LEU291, TRP301
Geranyl acetate	−6.2	ALA264, VAL270, ARG277, GLU284, PHE286
*Ocimum tenuiflorum* [tulsi]		
Baicalin	−9.6	GLN260, GLN281,THR282, GLY284
Kaempferol-3-o-Glucuronide	−9.2	GLN260, PHE314, THY333
Kaempferol	−9.1	PHE274, GLN281, LEU291

Eudesmol formed seven hydrophobic interactions (Pi-Alkyl) with the N protein, involving the amino acid residues Ala264 (3.86 and 6.06), Phe274 (5.86 and 6.01), and Trp301 (6.56, 5.25, and 7.47); two hydrogen bond interaction with Arg277 (5.33) and Phe286 (4.70) residues; and seven van der Waals interaction with Arg262, Thr263, Gly287, Val270, Leu291, Gly295, and Thr296, whereas linarin interacted with the N protein through six hydrogen bond interactions with residues *viz*., Phe274 (3.96), Arg277 (6.25), Thr282 (5.14), Gly284 (3.40 and 3.77), and Asn285 (3.75); three Hydrophobic interactions (Pi–Pi Stacked and Pi–Pi T-shaped) with Phe274 (4.65) and Phe286 (5.28 and 4.14) residues; and some van der Waals interactions with Gln281, Gln283, Trp301, Ile304, Tyr333, Leu353, and Ile357. (−)−Gamma−cadinene−N-protein complex showed eleven hydrophobic interactions (Pi-Alkyl) with Ala264 (5.99), Val270 (4.97, 4.96, and 6.01), Phe274 (4.47, 5.32, and 5.52,), Leu291 (4.41), and Trp301 (6.32, 7.38, 6.85, and 6.16), and six van der Waals interaction with Arg261, Thr263, Arg277, Phe286, Gly295, and Thr296. No hydrogen bonds were imputed between linarin and the binding site of the N protein (Figure 1).

### Phytoconstituents From *Coriandrum sativum* With the N Protein

**FIGURE 2:**
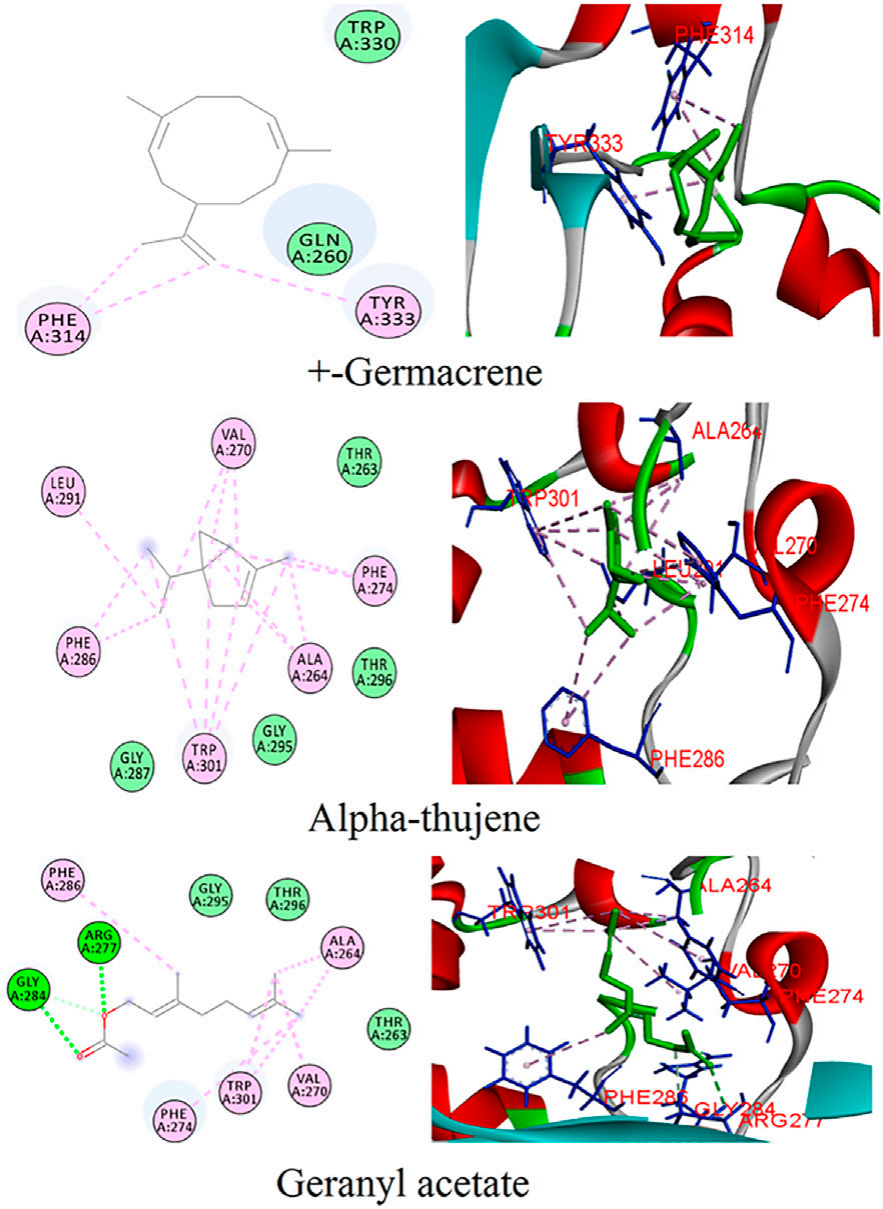
Binding patterns of the top phytoligands from *Coriandrum sativum* against the N protein.

The phytoligands of *Coriandrum sativum* were virtually screened against the N protein, and the docked phytoligands were ranked based on a stringer filter which included bonding affinity, strength of hydrogen bonding, and other hydrophobic, electrostatic, and van der Waals interaction. Out of 38 phytoligands, the top most docking poses and binding orientation were selected. The top ranked phytoligands include (+)−germacrene A, alpha-thujene, and geranyl acetate, which bind firmly at the active site of the target protein with high binding affinity and good molecular interactions (Table 1) (Figure 2). (+)−Germacrene A was bound to the N protein with the docking score of −7.1 kcal/mol, forming three hydrophobic interactions: (Pi-Alkyl) with Phe314 (5.35 and 5.50) and Tyr333(5.63), and two van der Waals interaction with Gln260 and Trp330. Alpha-thujene bound to the active site of the N protein with a docking score of 6.4 kcal/mol, and it displayed fifteen hydrophobic interactions (Pi-Alkyl) with Ala264 (5.51, 4.65, and 4.12), Val270 (4.78, 4.93, and 4.98), Phe274 (6.66 and 6.43), Phe286 (4.65 and 4.53), Leu291 (4.67), and Trp301 (6.55, 6.11, 5.36, and 6.68), and van der Waals interaction with residues Thr263, Gly287, Gly295, and Thr296. Geranyl acetate bound effectively to the active site of the N protein with the docking score of 6.2 kcal/mol, and it formed three hydrogen bonds (two conventional and one carbon hydrogen bond) with Arg277 (5.48) and Glu284 (3.48 and 3.91), respectively. Together, geranyl acetate also extended seven hydrophobic interactions (Pi-Alkyl) with resides Ala264 (3.74 and 4.06), Val270 (5.58), Phe274 (6.61), Phe286 (4.08), and Tro301 (5.37 and 6.62), and van der Waals interaction with Thr263, Gly295, and Thr296 residues (Figure 2).

### Binding Mode of Phytoconstituents From *Ocimum sanctum* With the N Protein

**FIGURE 3:**
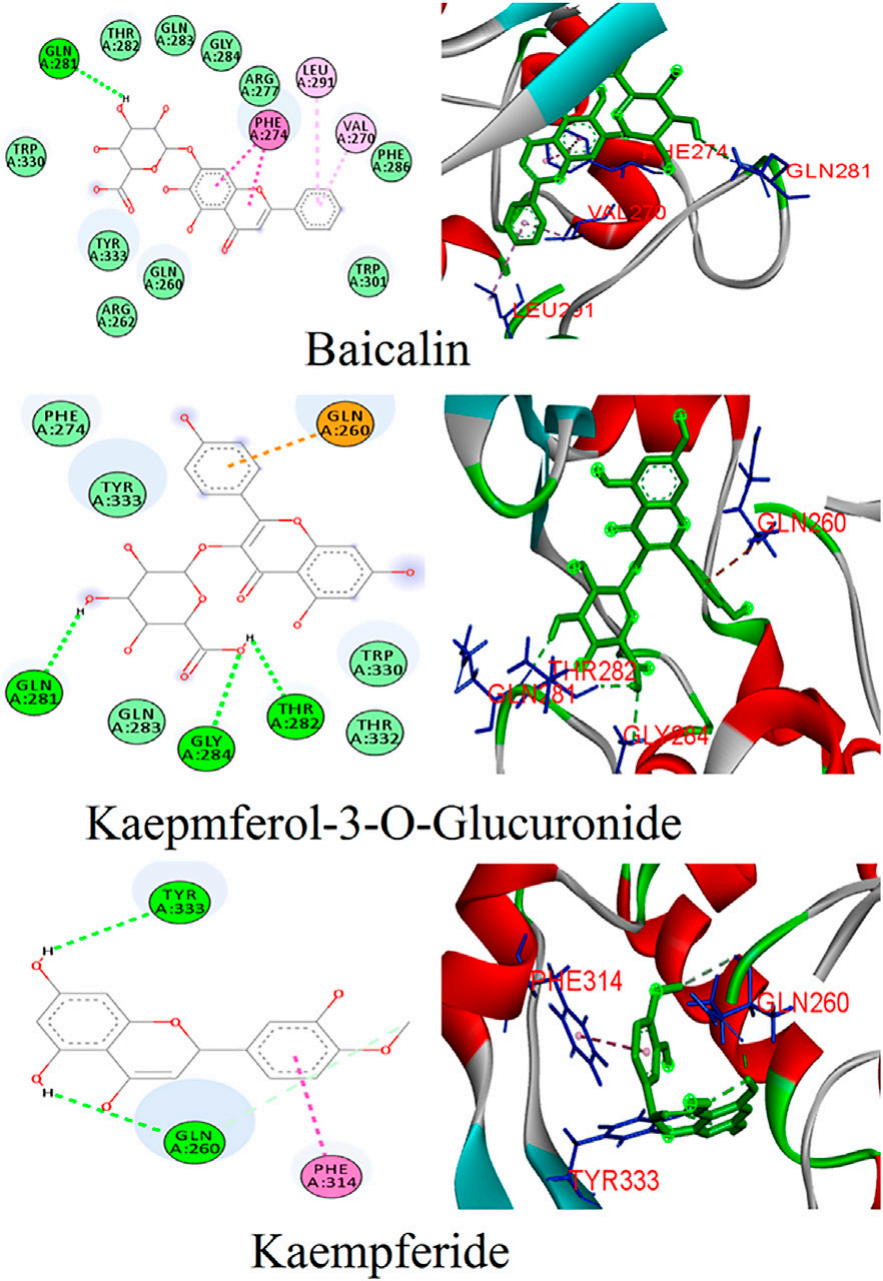
Binding patterns of the top phytoligands from *Ocimum tenuiflorum* against the N protein.

Twenty-three phytoligands from the immune booster herb *Ocimum tenuiflorum* were docked into the binding pocket of the N protein. After docking, three best phytoligands were selected based on their accurate binding pose and binding energy score. The top hit phytoligands were ranked in the sequence of baicalin, kaempferol-3-O-glucuronide, and kaempferide as they depicted a bonding affinity of −9.6, −9.2, and −9.1 kcal/mol, respectively (Table 1). Baicalin formed hydrogen bonds as well as hydrophobic interaction with the active site residues of receptor (Figure 3). It formed one hydrogen bond with the active site residue GLn281 (5.80); four hydrophobic interactions (Pi-alkyl, Pi–Pi stacked) with the active site residues of Val270 (5.24), Leu291 (7.32), and Phe274(4.85, 4.42); and some van der Waals interactions with resides Gln260, Arg261, Arg277, Thr282, Gln283, Gly284, Phe286, Trp301, Trp330, and Tyr333 (Figure 3). Kaempferol-3-O-glucuronide was stabilized by the three hydrogen bonds with active site residues of Gln281 (4.42), Thr282 (5.27), and Gly284 (3.73). One electrostatic interaction (Pi-cation) with the amino acid residue of Gln260 and some van der Waals interactions with amino acids residues of Phe274, Gln283, Trp330, Thr332, and Tyr333 were formed. Kaempferide was stabilized by forming three hydrogen bond interactions with active site residues of Gln260 (3.00 and 4.66) and Tyr333 (6.20), and one hydrophobic interaction (Pi–Pi stacked) with active site residue Phe314 (6.57) (Figure 3). A known antiviral drug nucleozin (which has been reported to target the N protein of influenza virus) was used as the positive control, which displayed a binding energy of −6.8 kcal mol^−1^ (Supplementary Table S1), and it builds one hydrogen bond with Ala264 (2.69Å); seven hydrophobic interactions (Pi–Pi stacked, Pi-alkyl, Pi-sigma, and Pi–Pi T-shaped) with amino acid residues Ala264 (4.52 Å), Val270 (3.93 Å), Phe286 (4.51 Å), Leu291 (5.33 Å), Trp301 (4.61 Å), Ile304 (5.35 Å), and Ala308 (3.66 Å); and van der Waals interaction with Arg262, Thr263, Arg277, Phe274, GLy295, Thr296, Ala305, Phe307, Leu353, and Ile357 (Supplementary Figure S1).

Overall, the docking results revealed that every docked complex formed fair number of Pi-alkyl, Pi–Pi stacked, Pi–Pi T-shaped, and Pi-cation interactions, which were largely involved in charge transfer that aid in intercalating the small molecules (drug) in the active site of the receptor (Arthur and Uzairu, 2019). The top hit phytoligands from each of three herbal plants displayed strong hydrophobic interactions and hydrogen bonds, which stabilized strong chemical bonding between phytoligands and the active site of the N protein.

### Drug-Likeness and ADME/T Prediction

Physicochemical properties, biological activity, and pharmacological profiles including absorption, distribution, metabolism, elimination, and toxicity (ADMET) features were envisaged using Molinspiration and admetSAR web server. The ADMET properties are essential in current drug discovery and development process. Nowadays, computational modeling is used instead of *in vitro* and *in vivo* evaluation of ADMET properties. The ultimate goal of *in silico* analysis is the perfect prediction of the *in vivo* pharmacokinetics of a probable drug molecule. Molinspiration results unveiled that all top phytoligands have obeyed Lipinski “Rule of 5” principles such as molecular weight (MW < 500Da), high lipophilicity expressed as logP (logP < 5), hydrogen bond donors (HBD < 5), and hydrogen bond acceptors (HBA < 10) (Table 2). Pharmacological parameters *viz*., blood–brain barrier penetration, human intestinal absorption, CYP2D6 inhibitor, Caco-2 cell permeability, carcinogenicity, and biodegradation of top phytoligands are depicted in Table 3. The very essential of ADMET property is the aqueous solubility of a drug, predicating the rate of absorption and transport of a drug molecule in the body and Caco-2 permeability, as it is one of the most important properties to measure the rate of transport of a drug molecule across the Caco-2 cell line. In addition, the BBB is a crucial factor for drugs, as it is a physiological barrier which protects the drug molecules to cross from blood to the brain (Alagu Lakshmi et al., 2020). In the present study, the ADMET prediction analysis revealed that the top hit phytoligands have the capability to aqueous solubility, Caco-2 permeability, cross the BBB, and novel absorption in the intestine. Therefore, the phytoligands envisaged in the current investigation could plausibly be considered as drug candidates for further studies.

**TABLE 2:** Pharmacodynamic profile of top three hit phytochemicals from each of the three selected plants (*Mentha arvensis*, *Coriandrum sativum*, and *Ocimum tenuiflorum*).

	Properties	Eudesmol	Linarin	(−)−Gamma-cadinene	(+)-Germacrene A	Alpha-thujene	Geranyl acetate	Baicalin	Kaempferol-3-O-glucuronide	Kaempferide
Absorption	Blood–brain barrier	BBB+	BBB−	BBB+	BBB+	BBB+	BBB+	BBB−	BBB−	BBB−
	Human intestinal absorption	HIA+	HIA+	HIA+	HIA+	HIA+	HIA+	HIA+	HIA+	HIA+
	Caco-2 permeability	Caco2+	Caco2−	Caco2+	Caco2+	Caco2+	Caco2+	Caco2−	Caco2−	Caco2+
	Renal organic transporter	Non-inhibitor	Non-inhibitor	Non-inhibitor	Non-inhibitor	Non-inhibitor	Non-inhibitor	Non-inhibitor	Non-inhibitor	Non-inhibitor
	Aqueous solubility (LogS)	−3.6183	−2.5665	−5.3703	−4.9808	−4.1737	−3.6996	−3.4620	−3.4620	−3.2219
Distribution	Subcellular localization	Lysosome	Mitochondria	Lysosome	Lysosome	Lysosome	Mitochondria	Mitochondria	Mitochondria	Mitochondria
Metabolism	CYP450 2C9 Substrate	Non-substrate	Non-substrate	Non-substrate	Non-substrate	Non-substrate	Non-substrate	Non-substrate	Non-substrate	Non-substrate
	CYP450 1A2 inhibitor	Non-inhibitor	Non-inhibitor	Non-inhibitor	Non-inhibitor	Non-inhibitor	Non-inhibitor	Non-inhibitor	Non-inhibitor	Non-inhibitor
	CYP450 2D6 inhibitor	Non-inhibitor	Non-inhibitor	Non-inhibitor	Non-inhibitor	Non-inhibitor	Non-inhibitor	Non-inhibitor	Non-inhibitor	Non-inhibitor
Toxicity	Human ether-a-go-go-related Gene inhibition	Weak inhibitor	Weak inhibitor	Weak inhibitor	Weak inhibitor	Weak inhibitor	Weak inhibitor	Weak inhibitor	Weak inhibitor	Weak inhibitor
	AMES toxicity	Non AMES toxic	Non-AMES toxic	Non-AMES toxic	Non-AMES toxic	Non-AMES toxic	Non-AMES toxic	Non-AMES toxic	Non-AMES toxic	Non-AMES toxic
	Carcinogens	Noncarcinogens	Noncarcinogens	Noncarcinogens	Noncarcinogens	Noncarcinogens	Carcinogens	Noncarcinogens	Noncarcinogens	Non-carcinogens
	Acute oral toxicity	III	III	III	III	III	III	II	II	III
	Rat acute toxicity	1.8911	2.6036	1.8911	1.5595	1.5330	1.5219	2.7357	2.7357	2.7192
	Fish toxicity	−0.3218	0.9183	−0.3218	−0.7436	−0.3500	0.2099	0.5766	0.5766	0.6628

**TABLE 3:** *In silico* drug-likeness and molecular property prediction in top three hit phytochemicals from each of the three selected plants (*Mentha arvensis*, *Coriandrum sativum*, and *Ocimum tenuiflorum*).

Phytoligands	MW	HBD	HBA	Log p[<5]	TPSA	nRO	nViol
Endesmol	222.37	1	1	4.01	20.23	1	0
Linarin	519.55	7	14	0.51	217.98	7	3
(−)−Gamma-cadinene	204.36	0	0	5.75	0.00	1	1
(+)−Germacrene A	204.36	0	0	5.46	0.00	1	1
Alpha-thujene	136.24	0	0	3.31	0.00	1	0
Geranyl acetate	196.29	0	0	3.91	26.30	6	0
Baicalin	187.12	6	11	0.55	187.12	4	2
Kaempferol-3-O-glucuronide	462.36	7	12	0.00	207.35	4	2
Kaempferide	300.27	3	6	2.71	100.13	2	0

MW: molecular weight, HBD: hydrogen bond donor, HBA: hydrogen bond acceptor, Log p, TPSA: total polar surface area, nRO: number of rotatable bond, nViol: number of violation.

### Molecular Dynamic Simulation

**FIGURE 4:**
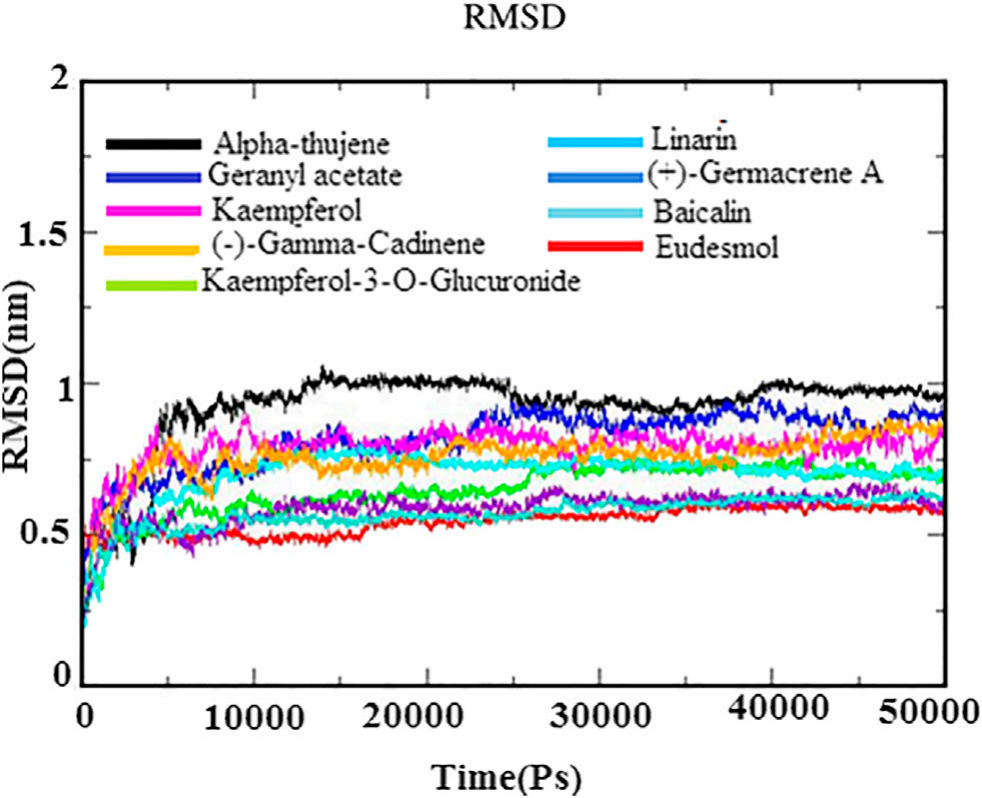
RMSD backbone plot for N-protein–inhibitor complexes (nine complexes) during 50 ns simulation as a function of timescale in ps.

MD simulations for protein–ligand complexes were performed for 50 ns. MD simulation is one of the attractive approaches to investigate the stability and dynamic behavior of the protein–ligand complexes in different binding poses under different physiological conditions. The observations of Cα backbone RMSD graph of docked complexes of each system suggested their stability during the simulation time period (Figure 4). Further, the RMSD graph also revealed that docked complexes were highly stable between 0.25 and 1.0 nm with minor deviations. The phytoligands *viz*., eudesmol, (+)−germacrene A, and baicalin acquired stability with an average RMSD of 0.5 nm, which depicted the stability of the protein–phytoligand complex in the active site of receptor. The phytoligands linarin geranyl acetate, kaempferol-3-O-glucuronide, and kaempferol (−)−gamma−cadinene showed slight deviation of RMSD around 0.65–0.8 nm. Alpha-thujene depicted more deviations at 1.0 nm; however, after 25 ns, it maintained the stability until 50 ns simulation. The RMSD analysis of the N protein with top phytoligands displayed that each phytoligand remained stable in the active site of the N protein throughout simulation (Figure 4).

**FIGURE 5:**
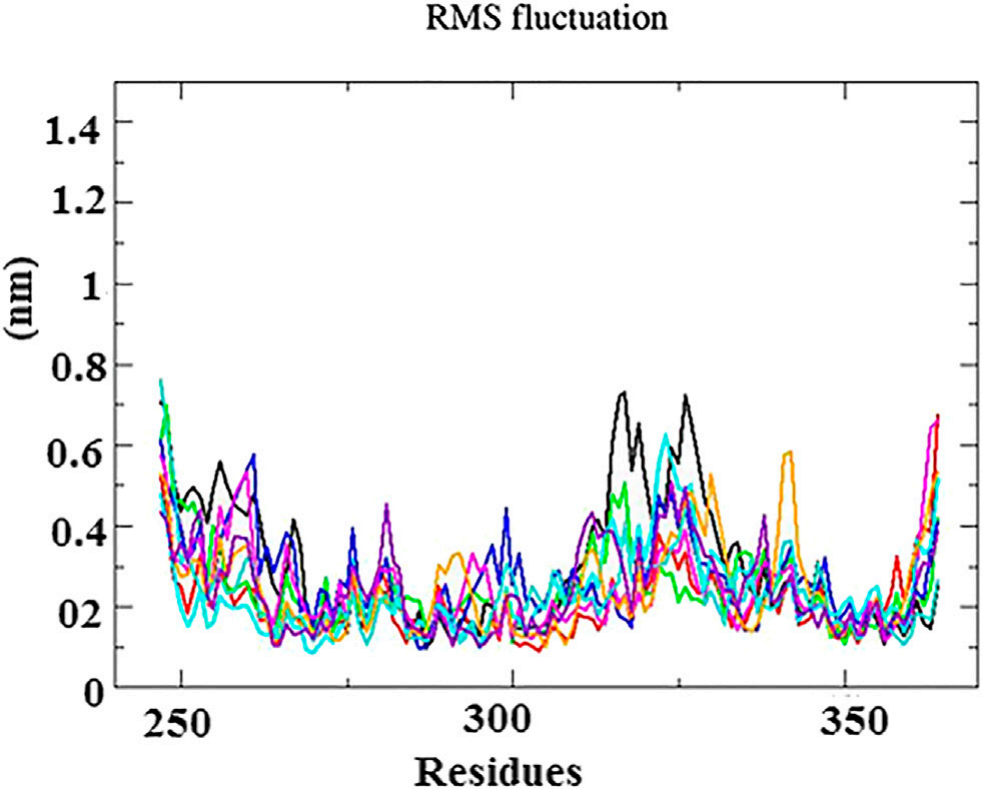
RMS fluctuation plot for N-protein–inhibitor complexes (nine complexes) during the 50 ns simulation as a function of the number of residues.

The root mean square fluctuation (RMSF) graphs of Cα backbone atom was used to study the dynamic behavior of essential amino acid residues precipitated with the ligand. As shown in Figure 5, the RMSF within the range of 0.4–1.8 Å had less structural fluctuations on interacting residues. Although high fluctuations were observed between the residues of 315–330, 335–340, and 364, these regions were denoted as the loop and disordered. Hence, the fluctuation does not affect the binding of ligand into the active site of protein.

**FIGURE 6:**
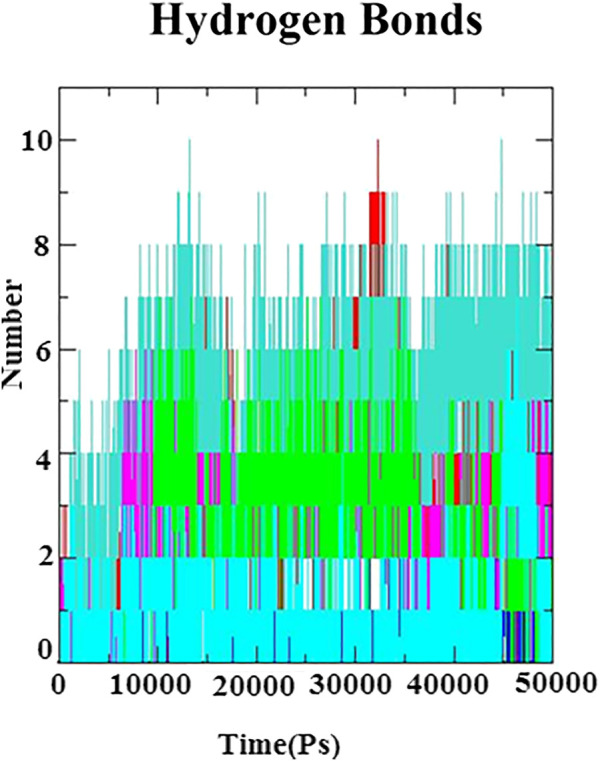
Hydrogen bond plot for N-protein–inhibitor complexes (nine complexes) during the 50 ns simulation as a function of timescale in ps.

In ligand–receptor (proteins) interaction, the role of hydrogen bond is inevitable for the molecular recognition, binding stability, and backbone conformation. Therefore, in order to assess the stabilizing interaction factor between the docked complexes, the number of hydrogen bonds was calculated to investigate the nature of the H-bond at the active site of the N protein. The H-bonds were monitored throughout 50 ns of simulation and is depicted in Figure 6. A maximum number of hydrogen bonds (*n* = 10) were identified with the complexes of the N protein and eudesmol as well as baicalin. Next to these, (−)−gamma−cadinene showed 8 h-bonds, linarin and kaempferol-3-O-glucuronide showed 7 H-bonds, (+)−germacrene A and geranyl acetate have shown 6 H-bonds, kaempferol showed 5H-bonds, and alpha-thujene showed 3H-bonds. All the H-bonds were stable and consistent throughout the 50-ns simulation.

## Conclusion

Concomitantly, in the current study, we envisaged nine (linarin, eudesmol, cadinene, geranyl acetate, alpha-thujene, germacrene A, kaempferol-3-O-glucuronide, kaempferide, and baicalin) phytochemicals out of 127 screened from three Indian herbal medicinal plants (*viz*., *Mentha arvensis*, *Coriandrum sativum*, and *Ocimum sanctum*) to target the N protein by exhibiting high binding affinity toward its catalytic pocket. Although a plethora of studies have targeted several other viral proteins (*viz*., spike protein, main protease, and receptor protein), the present study is first of its kind in envisaging phytochemicals against the N protein of SARS-CoV-2. Moreover, the data of ADMET prediction analysis depicted the nontumorigenic, noncarcinogenic, nontoxic, nonmutagenic, and nonreproductive nature of the identified bioactive molecules. Furthermore, the molecular dynamic simulation analysis validated the conformational and dynamic stability of the docked complexes. Overall, the data of the present study virtually authenticated the anti-COVID efficacy of phytochemicals from selected medicinal herbs of Indian origin.

## Data Availability

The original contributions presented in the study are included in the article/Supplementary Material; further inquiries can be directed to the corresponding authors.
