# Supplementary figures and images for: Indian Ethnomedicinal Phytochemicals as Promising Inhibitors of RNA-Binding Domain of SARS-CoV-2 Nucleocapsid Phosphoprotein: An In Silico Study
Source: Front Mol Biosci. 2021 Jul 2;8:637329. doi: 10.3389/fmolb.2021.637329 (PMC8283196; doi:10.3389/fmolb.2021.637329)

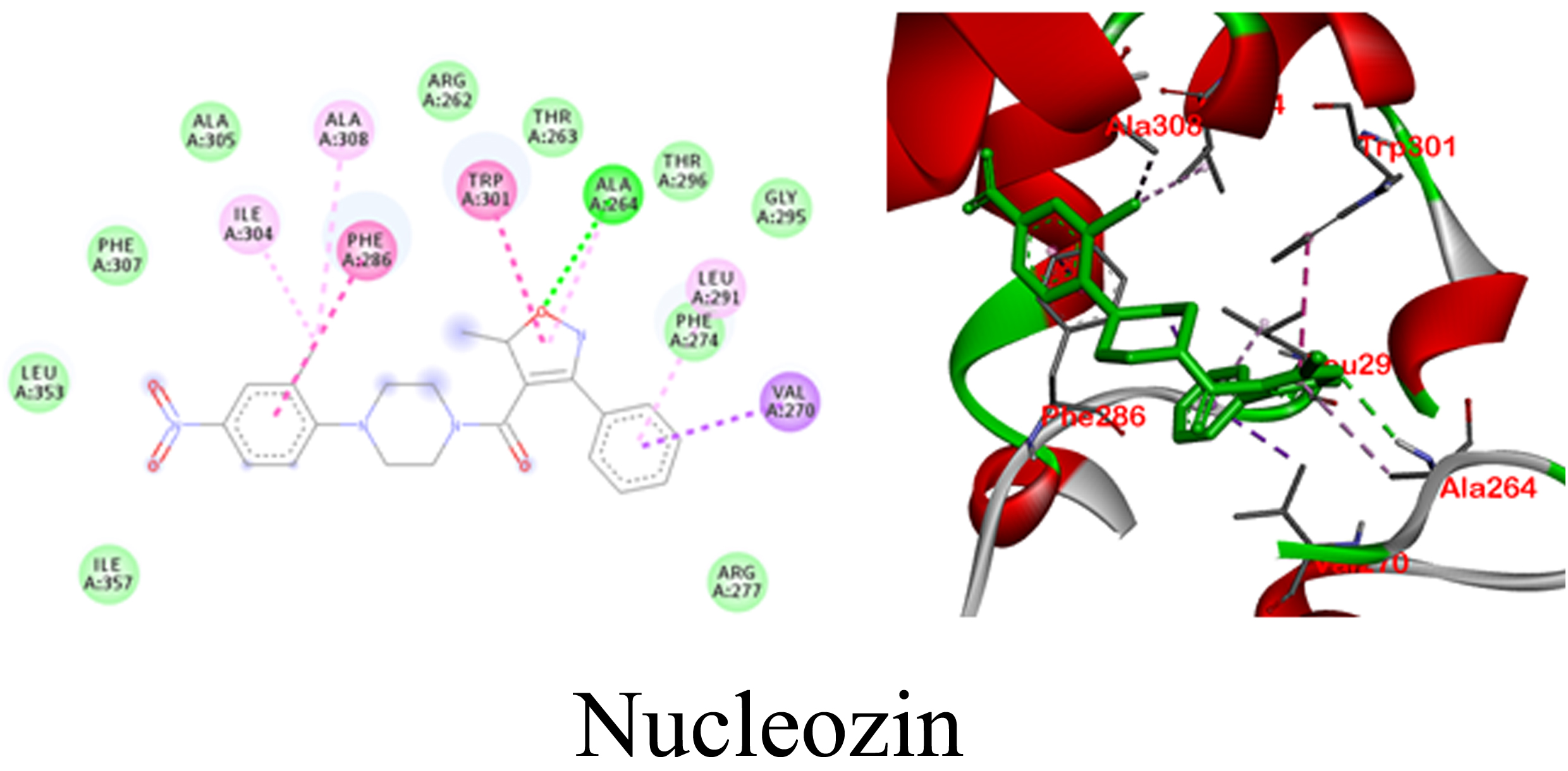

Supplement: Supplementary file 1 [file Image1.JPEG]
