# Supplementary material for: Indian Ethnomedicinal Phytochemicals as Promising Inhibitors of RNA-Binding Domain of SARS-CoV-2 Nucleocapsid Phosphoprotein: An In Silico Study
Source: Front Mol Biosci. 2021 Jul 2;8:637329. doi: 10.3389/fmolb.2021.637329 (PMC8283196; doi:10.3389/fmolb.2021.637329)
Supplement: Supplementary file 2 [file Table1.doc]

**Supplementary Table 1.** Binding interaction map of known SARS-CoV-2 protease inhibitor -lopinavir with SARS-CoV-2 3CL^pro^ **(A)** and Spike protein **(B)**. Active site residues in binding pockets are represented in three letter amino acid code and different types of interactions are denoted in different colours.

| **Sl. No.** | **Compound ID** | **Name of the phytochemical** | **Binding Affinity** |
| --- | --- | --- | --- |
| ***Mentha arvensis*** (Mint) | | | |
|  | 102024 | Cis-Carvyl Acetate | -6.0 |
|  | 10582 | Myrtenol | -5.5 |
|  | 11006 | Hexadecane | -5.1 |
|  | 11230 | 4-Carvomenthenol | -5.7 |
|  | 11463 | Alpha-Terpinolene | -6.1 |
|  | 11527 | Ethylamylcarbinol | -4.3 |
|  | 12216 | 3-Nonanol | -4.6 |
|  | 122484 | 2-Cyclohexen-1-Ol | -5.5 |
|  | 12302222 | Epi-Cadinol | -6.9 |
|  | 14896 | Beta-pinene | -5.6 |
|  | 1549108 | (2Z,6E)-Farnesol | -6.1 |
|  | 1549992 | Bisabolol | -6.8 |
|  | 1550884 | Alpha-Hexylcinnamaldehyde | -6.1 |
|  | 16666 | L-Menthol | -5.6 |
|  | 170833 | Isopulegol | -5.6 |
|  | 17100 | Alpha-Terpineol | -5.7 |
|  | 1742210 | Caryophyllene Oxide | -6.8 |
|  | 18818 | Thujene | -5.4 |
|  | 22311 | Limonene | -5.9 |
|  | 227829 | Guaiol | -7.2 |
|  | 26447 | (-)-Menthone | -5.8 |
|  | 2758 | Eucalyptol | -5.4 |
|  | 27867 | Menthyl Acetate | -5.9 |
|  | 29025 | Verbenone | -5.9 |
|  | 381152 | Piperitenone | -6.1 |
|  | 439263 | (+)-Neomenthol | -5.6 |
|  | 442495 | (+)-Pulegone | -6.0 |
|  | 525028 | 6-N-Heptadecene | -5.1 |
|  | 5281516 | Alpha-Farnesene | -6.6 |
|  | 5281520 | Humulene | -6.9 |
|  | 5317025 | Linarin | -8.4 |
|  | 5317319 | Cis-Beta-Farnesene | -6.2 |
|  | 5317570 | (-)-Germacrene D | -7.2 |
|  | 5352470 | Humulene Epoxide | -6.9 |
|  | 5354499 | L-Caryophyllene | -6.9 |
|  | 8164 | Octyl Acetate | -4.8 |
|  | 8174 | 1-Decanol | -4.6 |
|  | 8748 | Beta-terpineol | -5.6 |
|  | 8842 | Citronellol | -4.9 |
|  | 8914 | 1-Nonanol | -4.6 |
|  | 91457 | Eudesmol | -10.1 |
|  | 92313 | (-)-Gamma-Cadinene | -7.5 |
|  | 94221 | (-)-Trans-Carveol | -5.8 |
|  | 94266 | Sabinyl Acetate | -5.8 |
|  | 957 | 1-Octanol | -4.6 |
|  | 6432404 | (+)-Gamma-Cadinene | -7.5 |
|  | 6432469 | (-)-Isomenthone | -5.9 |
|  | 6441391 | Curdione | -6.9 |
|  | 6549 | Linalool | -5.2 |
|  | 6616 | Camphene | -5.5 |
|  | 6986 | DL-Menthone | -5.8 |
|  | 70247 | 1-Methoxyoctane | -4.3 |
|  | 7127 | Methyl Eugenol | -5.6 |
|  | 7439 | Carvone | -6.2 |
|  | 7461 | Gamma-terpinene | -5.8 |
|  | 7462 | Alpha-terpinene | -5.8 |
|  | 7463 | P-Cymene | -5.9 |
|  | 5363388 | Cis-3-Hexenyl Acetate | -4.7 |
|  | 5367681 | Cis-3-Hexenyl Isovalerate | -5.1 |
|  | 53677170 | (+)-Menthyl 4-Hydroxyphenylacetate | -7.4 |
|  | 61130 | Myrtenal | -5.8 |
|  | 61455 | Hexyl Isovalerate | -4.8 |
|  | 62367 | Sabinene Hydrate | -5.7 |
|  | 62566 | Beta-Bourbonene | -7.2 |
|  | 6428468 | Inosine-5'-Monophosphate | -6.1 |
|  | 6431302 | .Alpha.-Cadinol | -7.2 |
| (***Coriandrum sativum***) Coriander | | | |
|  | 10472 | 1-Tetracosanol | -5.3 |
|  | 121780 | 4-Dodecenal | -4.8 |
|  | 12404 | Arachidic Alcohol | -5.3 |
|  | 12512251 | (R)-2-Undecanol | -4.9 |
|  | 14896 | Beta-pinene | -5.6 |
|  | 15448 | 2-Undecanol | -5.1 |
|  | 1549026 | Geranyl acetate | -6.2 |
|  | 17868 | Alpha-thujene | -6.5 |
|  | 18818 | Sabinen | -5.4 |
|  | 22311 | Limonene | -6.0 |
|  | 25311 | Tridecanal | -4.7 |
|  | 2537 | Camphor | -5.5 |
|  | 2758 | Eucalyptol | -5.4 |
|  | 31253 | Myrcene | -5.3 |
|  | 31291 | Tetradecanal | -5.0 |
|  | 440917 | D-Limonene | -5.9 |
|  | 5280541 | (E)-2-Hexadecenal | -5.5 |
|  | 5283345 | Trans-2-Decenal | -4.7 |
|  | 5283356 | Trans-2-Undecenal | -5.0 |
|  | 5283361 | Trans-2-Dodecenal | -4.7 |
|  | 5283363 | 2-Tridecenal | -5.2 |
|  | 5283366 | 2-Tetradecenal | -5.5 |
|  | 5355330 | DiallylFumarate | -5.3 |
|  | 537338 | Heptadecenal | -5.4 |
|  | 637566 | Geraniol | -5.5 |
|  | 6428898 | 2-Pentadecenal | -5.5 |
|  | 6549 | Linalool | -5.2 |
|  | 6616 | Camphene | -5.3 |
|  | 6654 | Alpha-pinene | -5.7 |
|  | 7439 | Carvone | -6.0 |
|  | 7461 | Gamma-terpinene | -5.0 |
|  | 8141 | Nonane | -4.6 |
|  | 8174 | 1-Decanol | -4.9 |
|  | 8175 | Decanal | -4.7 |
|  | 8186 | Undecanal | -5.0 |
|  | 8194 | Dodecanal | -4.8 |
|  | 8201 | Methyl stearate | -5.8 |
|  | 9548705 | (+)-Germacrene A | -7.1 |
| (***Ocimum tenuiflorum***) Tulsi | | | |
|  | 1454027 | carbonic anhydrase 2 | -8.7 |
|  | 13283 | 4,4'-Methylenebis(2-Methylaniline) | -8.2 |
|  | 160481 | Isosakuranetin | -8.0 |
|  | 160921 | Nevadensin | -8.0 |
|  | 22846027 | Kaempferol-3-O-Glucuronide | -9.2 |
|  | 3026 | Dibutyl Phthalate | -6.1 |
|  | 40326 | Permethrin | -8.8 |
|  | 441773 | Peonidin | -8.1 |
|  | 5280443 | Apigenin | -8.3 |
|  | 5280445 | Luteolin | -8.1 |
|  | 5280863 | Kaempferol | -7.9 |
|  | 5280961 | Genistein | -8.2 |
|  | 5281612 | Diosmetin | -8.2 |
|  | 5281666 | Kaempferide | -9.1 |
|  | 5281792 | Rosmarinic Acid | -7.8 |
|  | 60961 | Adenosine | -6.4 |
|  | 64982 | Baicalin | -9.6 |
|  | 689043 | Caffeic Acid | -6.1 |
|  | 717531 | Dimethyl Caffeic Acid | -6.0 |
|  | 73207 | Xanthomicrol | -7.8 |
|  | 735755 | 3,4,5-Trimethoxycinnamic acid | -6.0 |
|  | 74315894 | Peonidin-3-O-Alpha-Arabinopyranoside | -8.8 |
|  | 8343 | Bis(2-Ethylhexyl) Phthalate | -6.9 |
|  | 2863945 | Nucleozin (positive control) | -6.8 |
