# Supplementary material for: Indian Ethnomedicinal Phytochemicals as Promising Inhibitors of RNA-Binding Domain of SARS-CoV-2 Nucleocapsid Phosphoprotein: An In Silico Study
Source: Front Mol Biosci. 2021 Jul 2;8:637329. doi: 10.3389/fmolb.2021.637329 (PMC8283196; doi:10.3389/fmolb.2021.637329)
Supplement: Supplementary file 3 [file Table3.DOCX]

Table 3:

| **Phytoligands** | **MW** | **HBD** | **HBA** | **Log p[<5]** | **TPSA** | **nRO** | **nViol** |
| --- | --- | --- | --- | --- | --- | --- | --- |
| Endesmol | 222.37 | 1 | 1 | 4.01 | 20.23 | 1 | 0 |
| Linarin | 519.55 | 7 | 14 | 0.51 | 217.98 | 7 | 3 |
| (-)-Gamma-Cadinene | 204.36 | 0 | 0 | 5.75 | 0.00 | 1 | 1 |
| (+)-Germacrene A | 204.36 | 0 | 0 | 5.46 | 0.00 | 1 | 1 |
| Alpha-thujene | 136.24 | 0 | 0 | 3.31 | 0.00 | 1 | 0 |
| Geranyl acetate | 196.29 | 0 | 0 | 3.91 | 26.30 | 6 | 0 |
| Baicalin | 187.12 | 6 | 11 | 0.55 | 187.12 | 4 | 2 |
| Kaempferol-3-O-Glucuronide | 462.36 | 7 | 12 | 0.00 | 207.35 | 4 | 2 |
| Kaempferide | 300.27 | 3 | 6 | 2.71 | 100.13 | 2 | 0 |

MW-molecular weight, HBD-hydrogen bond donor, HBA- hydrogen bond acceptor, Log p, TPSA-Total polar surface area, nRO-Number of rotatable bond, nViol-Number of violation.
