# Supplementary material for: Indian Ethnomedicinal Phytochemicals as Promising Inhibitors of RNA-Binding Domain of SARS-CoV-2 Nucleocapsid Phosphoprotein: An In Silico Study
Source: Front Mol Biosci. 2021 Jul 2;8:637329. doi: 10.3389/fmolb.2021.637329 (PMC8283196; doi:10.3389/fmolb.2021.637329)
Supplement: Supplementary file 4 [file Table4.docx]

| **Compound name** | **Binding affinity** | **Interaction residues** |
| --- | --- | --- |
| **Interaction diagram of *Menthe arvensis* [mint]** | | |
| Eudesmol | -10.1 | ALA264, PHE274, ARG277, PHE286, TRP301 |
| Linarin | -8.4 | PHE274, ARG277, THR282, GLY284, PHE286 |
| (-)-Gamma-Cadinene | -7.5 | ALA264, VAL270, PHE271, LEU291, TRP301 |
| ***Coriandrum sativum* [coriander]** | | |
| (+)-Germacrene | -7.1 | PHE314, TYR333 |
| Alpha-thujene | -6.5 | ALA264, VAL270, PHE286, LEU291, TRP301 |
| Geranyl acetate | -6.2 | ALA264, VAL270, ARG277, GLU284, PHE286 |
| ***Ocimum tenuiflorum* [tulsi]** | | |
| Baicalin | -9.6 | GLN260, GLN281,THR282, GLY284 |
| Kaempferol-3-o-Glucuronide | -9.2 | GLN260, PHE314, THY333, |
| Kaempferol | -9.1 | PHE274, GLN281, LEU291 |
