# Supplementary material for: Indian Ethnomedicinal Phytochemicals as Promising Inhibitors of RNA-Binding Domain of SARS-CoV-2 Nucleocapsid Phosphoprotein: An In Silico Study
Source: Front Mol Biosci. 2021 Jul 2;8:637329. doi: 10.3389/fmolb.2021.637329 (PMC8283196; doi:10.3389/fmolb.2021.637329)
Supplement: Supplementary file 5 [file Table2.DOC]

**Table 2:**

|  | Properties | Eudesmol | Linarin | (-)-Gamma-Cadinene | (+)-Germacrene A | Alpha-thujene | Geranyl acetate | Baicalin | Kaempferol-3-O-Glucuronide | Kaempferide |
| --- | --- | --- | --- | --- | --- | --- | --- | --- | --- | --- |
| Absorption | Blood-Brain Barrier | BBB+ | BBB- | BBB+ | BBB+ | BBB+ | BBB+ | BBB- | BBB- | BBB- |
|  | Human Intestinal Absorption | HIA+ | HIA+ | HIA+ | HIA+ | HIA+ | HIA+ | HIA+ | HIA+ | HIA+ |
|  | Caco-2 Permeability | Caco2+ | Caco2- | Caco2+ | Caco2+ | Caco2+ | Caco2+ | Caco2- | Caco2- | Caco2+ |
|  | Renal Organic Transporter | Non-inhibitor | Non-inhibitor | Non-inhibitor | Non-inhibitor | Non-inhibitor | Non-inhibitor | Non-inhibitor | Non-inhibitor | Non-inhibitor |
|  | Aqueous solubility  (LogS) | -3.6183 | -2.5665 | -5.3703 | -4.9808 | -4.1737 | -3.6996 | -3.4620 | -3.4620 | -3.2219 |
| Distribution | Sub-cellular localization | Lysosome | Mitochondria | Lysosome | Lysosome | Lysosome | Mitochondria | Mitochondria | Mitochondria | Mitochondria |
| Metabolism | CYP450 2C9 Substrate | Non-substrate | Non-substrate | Non-substrate | Non-substrate | Non-substrate | Non-substrate | Non-substrate | Non-substrate | Non-substrate |
|  | CYP450 1A2 Inhibitor | Non-inhibitor | Non-inhibitor | Non-inhibitor | Non-inhibitor | Non-inhibitor | Non-inhibitor | Non-inhibitor | Non-inhibitor | Non-inhibitor |
|  | CYP450 2D6 Inhibitor | Non-inhibitor | Non-inhibitor | Non-inhibitor | Non-inhibitor | Non-inhibitor | Non-inhibitor | Non-inhibitor | Non-inhibitor | Non-inhibitor |
| Toxicity | Human Ether-a-go-go-Related Gene Inhibition | Weak inhibitor | Weak inhibitor | Weak inhibitor | Weak inhibitor | Weak inhibitor | Weak inhibitor | Weak inhibitor | Weak inhibitor | Weak inhibitor |
|  | AMES Toxicity | Non AMES toxic | Non AMES toxic | Non AMES toxic | Non AMES toxic | Non AMES toxic | Non AMES toxic | Non AMES toxic | Non AMES toxic | Non AMES toxic |
|  | Carcinogens | Non- Carcinogens | Non- Carcinogens | Non- Carcinogens | Non- Carcinogens | Non- Carcinogens | Carcinogens | Non- Carcinogens | Non- Carcinogens | Non- Carcinogens |
|  | Acute Oral Toxicity | III | III | III | III | III | III | II | II | III |
|  | Rat Acute Toxicity | 1.8911 | 2.6036 | 1.8911 | 1.5595 | 1.5330 | 1.5219 | 2.7357 | 2.7357 | 2.7192 |
|  | Fish Toxicity | -0.3218 | 0.9183 | -0.3218 | -0.7436 | -0.3500 | 0.2099 | 0.5766 | 0.5766 | 0.6628 |
